# Accuracy vs. Practicality of Inertial Measurement Unit Sensors to Evaluate Motor Competence in Children

**DOI:** 10.3389/fspor.2022.917340

**Published:** 2022-06-15

**Authors:** Natalie Lander, Darius Nahavandi, Nicole G. Toomey, Lisa M. Barnett, Shady Mohamed

**Affiliations:** ^1^Faculty of Health, Institute for Physical Activity and Nutrition, Deakin University, Geelong, VIC, Australia; ^2^Institute for Intelligent Systems Research and Innovation, Deakin University, Geelong, VIC, Australia; ^3^Faculty of Health, School of Health and Social Development, Deakin University, Geelong, VIC, Australia

**Keywords:** TGMD, feasibility, accuracy, machine learning, schools, TGMD-3, accuracy, machine learning

## Abstract

The TGMD (i.e., Test of Gross Motor Development) has been considered as one of the gold standards of assessment tools for analysis of motor competence in children. However, it is rarely used by teachers in schools because the time, resources, and expertise required for one teacher to assess a class of students is prohibitive in most cases. A potential solution may be to automate the testing protocol using objective measures and inertial measurement unit sensors. An accurate method using 17 sensors to capture full body motion profiles and machine learning methods to objectively assess proficiency has been developed; however, feasibility of this method was low. Subsequently, a simplified method using four sensors (i.e., attached to wrists and ankles) was found to be effective, efficient, and potentially highly feasible for use in school settings. For some skills, however, not all skill criteria could be assessed. Additionally, misclassification on occasion, marred results. In the present paper we consider a previous experiment that used wireless motion capture to assess criteria from the TGMD-3. We discuss the advantages alongside the disadvantages of testing motor competence in children using sensors and consider the question—Can a compromise be struck between accuracy and feasibility?

## Introduction

### Assessing Motor Competence in Children

Physical activity is a positive contributing factor for child health and development (WHO, 2020). Current World Health Organization (WHO) guidelines recommend that young people (aged 5–17 years) should accumulate an average of 60 min of moderate-to-vigorous intensity physical activity daily to achieve related benefits (WHO, 2020). However, globally the proportion of children and adolescents meeting these recommendations is low (Chaput et al., 2020). Motor competence is one determinant of physical activity (Stodden et al., 2008; De Meester et al., 2020). Motor competence describes proficient performance of a broad range of motor skills as well as underlying mechanisms including quality of movement, motor coordination, and motor control (Robinson et al., 2015; Utesch and Bardid, 2019). Motor competence incorporates discrete fundamental movement skills such as throwing, catching, running, jumping, balancing, and twisting (Gallahue et al., 2012; Rudd et al., 2016), which are considered the foundation of more advanced movements used in sports and other physical activities (Clark and Metcalfe, 2002; Logan et al., 2018; Barnett et al., 2021).

In children and adolescents, low motor competence correlates with unhealthy weight status (Cattuzzo et al., 2016; Barnett et al., 2021), low health-related fitness (Utesch and Bardid, 2019), poor social and cognitive outcomes (Bremer and Cairney, 2018; Macdonald et al., 2018), and low levels of physical activity later in life (Stodden et al., 2008). Despite the substantial health benefits, levels of motor competence are low worldwide. Therefore, developing motor competence in children is a global research priority, and a central component of the physical education curriculum in schools (Poitras et al., 2016; Pingitore et al., 2019; Rodriguez-Ayllon et al., 2019). Indeed, there has been burgeoning international interest in monitoring, investigation, and interventions of motor competence of children and youth (Logan et al., 2018).

Early identification of children with low motor competence is an economically efficient and effective way to minimize physical developmental delays (Burton and Miller, 1998). Correct measurement of motor competence can assist planning, implementation, and evaluation for research-based interventions and school-based physical education programs. In schools, assessment of motor competence can help teachers identify deficits, plan for and deliver targeted teaching, and determine teaching and learning outcomes (Burton and Miller, 1998). Motor competence assessment tools can be beneficial (Vandorpe et al., 2011; Brian et al., 2019; Mombarg et al., 2021), of which there are many available (Griffiths et al., 2018; Scheuer et al., 2019). The Test of Gross Motor Development (TGMD) may be the most well-studied in research and clinical settings (Webster and Ulrich, 2017). The TGMD (Ulrich, 2017) is an internationally recognized process-based motor skill assessment for children aged 3–10 years (Ulrich, 2017; Webster and Ulrich, 2017). This assessment is now in its third iteration (TGMD-3), and the full assessment comprises 13 skills, each evaluated against several criteria and scored as 1 (i.e., correct) or 0 (i.e., incorrect). The results clearly and comprehensively indicate skill competence and pinpoint areas for remediation (Ulrich, 2017; Webster and Ulrich, 2017). However, although MC is a curriculum priority, motor competence is rarely assessed using instruments such as the TGMD, by teachers in school settings, and skill deficits often go undiagnosed (Lander et al., 2019).

Despite the advantages, the TGMD-3 has some potential limitations for wide-scale use, particularly in real-world settings such as school-based physical education. For example, the TGMD-3 can be intensive to administer (e.g., requires one-to-one administration with extended assessment duration) and time intensive to analyze (Wiart and Darrah, 2001; Steadward et al., 2003). Further, the criteria are complex and potentially difficult to interpret in real time or even via video assessment without specific training. Subsequently, the time, resources, and expertise required for one teacher to assess an entire class of students using the TGMD, is prohibitive in most cases. To address the current limitations of manual motor competence assessment in real-world settings, various technologies have been introduced to automate the process, using systems based on cameras, optical markers, or inertial measurement units. An inertial measurement unit (IMU) is an electronic device that measures a body's specific force, angular rate, and sometimes orientation. They have typically been used to maneuver aircraft (Bruggemann et al., 2011). Recently, IMUs have been used to investigate movement quality in children (Grimpampi et al., 2016; Clark et al., 2019). The IMU sensors are placed on different parts of the body and motion tracked by estimated kinematics of the joints through the direction and orientation of the IMU sensors while the subject is performing the skills (Lander et al., 2020). IMU-based systems are suitable for indoor and outdoor environments, and do not require complex or expensive equipment. The system is portable, lightweight, inexpensive, easily replaceable, and low maintenance. Accordingly, IMU-based systems may be well-suited to large-scale usage such as large numbers of students in school physical education classes.

As an alternative to manual assessment of the TGMD-3, IMUs have been used in a protocol to objectively assess seven TGMD-3 skills in children (Lander et al., 2020). Use of 17 sensors provided sufficient accuracy, but due to the time required to set up 17 sensors, it was considered potentially unfeasible for use by teachers in school physical education settings. In contrast, use of four sensors (i.e., positioned on the wrists and ankles) and automated analysis using machine learning, substantially reduced the assessment and analysis burden (e.g., time and expertise), and consequently made the assessment of motor competence more feasible (i.e., being more easily or conveniently done) in school physical education settings (Lander et al., 2020). However, concluding that the results of the study were reliable and accurate may have been overly optimistic. Indeed, there are some challenges regarding use of IMUs to accurately and comprehensively assess motor competence in school students. Therefore, the aim of the present paper was to use results from a previous study to examine the advantages and disadvantages of using IMU-based motion capture technology to automate the TGMD-3 for use in real-world school physical education settings.

## Methods

### Reconsidering the IMU Method

As an attractive alternative to manual assessment of the TGMD-3, advantages of IMUs include: less time to complete the assessment; no time required to analyze performances as it is automated; no need for specific assessor expertise for analysis of complex criteria as analysis is automated; simultaneous assessment of small groups of students; reliable and accurate assessment; and avoiding the ethical constraints of videoing children. The major advantage of an automated IMU method is that it is straightforward, time efficient and simple enough to be practical in the context of the classroom or physical education setting. However, there are also several disadvantages to streamlining the assessment process. In the present paper, data from the previous study (Lander et al., 2020) are reconsidered in greater detail. In particular, the inconsistent, missing, and weak data points were closely examined to consider extent of incompleteness and potential explanations and implications. For full details of the original experimental procedure, see the paper entitled “Bringing objectivity to motor skill assessment in children” by Lander et al. (2020). An overview of the methods are presented in brief below.

### Materials and Methods

In order to demonstrate the advantages and disadvantages of IMU-based motion capture technology for the purpose of automating the TGMD-3, a case study was used which captured seven of the TGMD-3 skills, as a sample (Lander et al., 2020). University ethics (blinded) and the Department for Education and Training (blinded) approved this research. Children from Melbourne, Australia, were recruited using convenience sampling. Interested parents/guardians were emailed a plain language statement and written consent form. All children had no known developmental delay and no musculoskeletal pathology. Participation only proceeded following signed parent/guardian consent and child assent. Parents/Guardians provided basic demographical details (i.e., child: date of birth and sex), by way of a survey at the time of consent. Fourteen children in total (nine boys and five girls) participated, the ages ranged from seven to 12 years (M = 9.64). The following section describes the experiments and the results.

### Motor Skill Performance—TGMD-3

Seven of the 13 skills TDMD-3 skills were performed by the children. This included the four locomotor skills (jump, hop, skip and side-step) and three object control skills (catch, throw and kick). These skills were selected as they are commonly used in the Australian physical education curriculum as well as junior sport and physical activity pursuits. Children performed the TGMD-3 in school or university gymnasiums suitable for the administration of the TGMD-3, and according to the test instructions. Before participant performance, an accurate demonstration of the skill was performed. Participants were tested individually and were given one practice trial to ensure that they understood what to do. Each participant performed one to 10 trials for each of the seven motor skills based on the need for variability to inform the sensor data. The video camera was placed optimally (i.e., side view, frontal view, or rear view) to best detect the particular skill performance for subsequent analysis and scoring.

### Motion Capture

In this case-study (Lander et al., 2020) the XSENS AWINDA motion capture system (*Xsens* 2022) was used. This is an inertial IMU-based motion capture system comprising of 17 wireless sensors with the ability to capture motion at a rate of 60 Hz. To configure and calibrate the kinematic model, a series of predefined bony landmarks were collected from participants. Measurement of these landmarks were collected with an anthropometric measuring tape. The measurements consisted of body height (measurement from ground to the top of head when standing upright), shoe size (measurement from top of shoe nose to the end of the heel), arm span (measurement from top of right fingers to top of the left fingers in T-Pose), hip height (measurement of ground to most lateral bony prominence of the greater trochanter), knee height (measurement of ground to lateral epicondyle on the femoral bone), ankle height (measurement of ground to distal tip of the lateral malleolus), hip width (measurement of right to the left anterior superior iliac spine), shoulder width (measurement of right to the left distal tip of acromion), shoe sole thickness (measurement of average thickness of the sole of the used shoes). Once participants' anthropometrics were collected, they were fitted with sensors via Velcro straps. Students wore these on top of their clothing tightly and secure to ensure no unexpected movement of sensors would take place. This included a shirt with fitted belt, leg straps (lower and upper leg), arm straps (forearm and upper arm), gloves, shoe covers, and a head band. The calibration of the motion capture suit within the XSENS MVN Analyze 2019 software was conducted prior to each capture to ensure accurate sensor data.

### Manual Assessment of Video and Motion Data

The video data collected of the participants was analyzed based on meeting the TGMD-3 skill criteria; and scored 1 or 0 based on whether the criterion was fulfilled or not (respectively). Those assessing the video data had completed online training/coding specifically for this assessment and achieved a reliability of >0.95 against expert raters (Lander et al., 2020). Each video was assessed multiple times at varying speeds and angles to ensure a correct score was given per each criterion. In addition, a manual visual observation was also performed on each of the motion data recordings. This involved a process of analyzing the motion data and parameters that could alter data such as sensor drift or bad calibration. Once a high standard of motion data was confirmed, the motion data was assessed via the kinematic human model on its performance to the TGMD-3. This provided a further mechanism of scoring criteria correctly for participants.

### Sensor Data Analysis

The full motion capture suit was worn by participants comprising of 17 IMU sensors. A full body set up was calibrated and utilized to enable a realistic full body viewing platform for visual analysis of the TGMD-3. However, in an attempt to bypass the need for the traditional manual visual observation of assessment, the raw signals from the sensors were extracted, including angular velocity and orientation. The raw signals from the sensors were further post-processed to extract the smallest number of features in time-domain and frequency-domain to achieve a high level of accuracy using Machine Learning models, as presented in detail in the previous paper (Lander et al., 2020). This technique proposes to highlight the sensors / sensor locations (i.e., the reduction from 17 to four sensors: right and left forearm and ankle) that provided insight to the overall motion profile of the participant for each skill and each skill criteria. Upon viewing of the motion path trajectories during motor skill performances, specific features of acceleration were defined for the algorithm. A benchmark was determined using a gold-standard performance of each skill as reference acceleration data for each criterion of the TGMD-3 (Lander et al., 2020). Features and similarities of performances were assessed against this gold-standard and automatically scored accordingly [see (Lander et al., 2020) for more detail]. The output was then reviewed against the manual scoring of the motion file to assess the reliability of the IMU-based algorithms.

## Results

Among skills that were satisfactorily assessed when reviewed against the manual scoring file, the skip and the side-step were both correctly classified 100% of the time. The kick and the catch had accuracy per skill of around 95% for elements that could be assessed and had very few misclassifications. The throw reached 80.5% accuracy, and the jump and hop were close to 80% accuracy [see (Lander et al., 2020) for full detail]. However, some skill criteria, or components of skill criteria, were not able to be assessed by the IMUs. Table 1 summarizes the TGMD-3 criteria that were confidently recognized by IMU-based systems, and the criteria or components within the criteria of each skill that had limited success according to the inability to assess these aspects via IMUs. The red font highlights aspects of the criteria that were not able to be assessed.

**Table 1:** The TGMD-3 skill criteria that can and cannot be assessed via IMUs.

**Skill**	**Skill criteria**	**Can be assessed using IMUs**
Hop	Non-support leg swings forward in pendula fashion to produce force	✓
	Foot of non-support leg remains behind body	✓
	Arms flexed and swing forward to produce force	✓
	Takes off and lands four consecutive times	✓
Sidestep	Body turned sideways so shoulders are aligned with line on floor	✓
	A step forward with lead foot followed by a slide of the trailing foot to a point next to the lead foot	✓
	A minimum of four continuous step slide cycles to the right	✓
	A minimum of four continuous step slide cycles to the left	✓
Skip	Step forward followed by a hop on same leg	✓
	Arms flexed and swinging forward	✓
	Four continuous hop cycles	✓
Catch	Hands out in front elbows flexed	✓
	Arms extend while reaching for the ball	*X*
	Catch ball with hands only	*X*
Kick	Rapid and continuous approach	✓
	Elongated step or leap prior to ball contact	✓
	Non-kicking foot close to ball	*X*
	Use instep or inside of preferred foot to kick ball—not toe	*X*
Jump	Preparatory movement includes flexion of both knees with arms extended behind body	*X*
	Arms extended forcefully forward and upward reaching full extension above the head	*X*
	Take off and land on both feet simultaneously	✓
	Arms are thrust downward during landing	✓
Overhand throw	Windup is initiated with downward movement of hand/arm	✓
	Rotates hip and shoulders to a point where the non-throwing side faces the wall	*X*
	Weight is transferred by stepping with the foot opposite the throwing hand	✓
	Follow-through beyond ball release diagonally across the body toward the non-preferred side	*X*

## Discussion

The aim of this paper was to examine the advantages and disadvantages of using IMU-based motion capture technology to automate the TGMD-3 for use in real-world school physical education settings. Findings demonstrated that although there are major advantages of using IMUs for motor competence assessment, there are also significant disadvantages. This section discusses the key advantages and disadvantages of IMU use, which need to be considered when making decisions about the application of MC assessments in real-world settings such as schools.

All four TGMD-3 criteria of the hop could be tracked by IMUs, all four criteria of the sidestep, and all three of the skip (see Table 1). Therefore, these three skills could be considered comprehensively assessed by the four-sensor IMU method. This is reflected in the 100% accuracy of the skip and sidestep. The hop achieved 80% accuracy. This compares favorably to manual assessment, where a review of TGMD reliability reported that whilst manual assessment of the skip showed generally acceptable levels (ICC ≥ 0.6) of inter-rater reliability, that this was not the case for the hop, horizontal jump, and slide. The same review also reported that intra-rater reliability was good for the skip and horizontal jump, but not for the hop or slide. Authors stated that differences in reliability between skills could be due to the challenges in assessing some performance criteria.

Further, the localization problem of the four IMU sensors also affects accuracy in estimating some movements, particularly obvious in a locomotor skill. For example, although the jump did not involve any external objects, such as a ball or wall, two of the four criteria could not be assessed due to localization, which concerned the relativity or positionality in regard to other body parts. A key component in the preparatory phase of the jump includes “flexion of both knees with arms extended *behind body*.” However, this could not be assessed with only four sensors on wrists and ankles, as the position on the arms in relation to the body could not be detected. Further, the arms should be “extended forcefully forward and upward reaching full extension above the head” during the jump. The IMU tracking algorithms are not able to capture the full extension of the arms because the four sensors do not allow accurate estimation of the position of the hands relative to the elbow. Thus, skill assessment is not comprehensive.

Therefore, the problem of localization is one of the main limitations of IMUs to assess TGMD-3 skills. While it is relatively simple to identify the attitude and accelerations of the IMU sensor, it is harder to identify position and distance traveled. Sensor fusion can be used to estimate the positioning of the sensor to a certain limit, but not to the level of accuracy required to assess motion against TGMD-3 criteria. Thus, aspects of some skill criteria were not measurable.

Limitations of the simplified, four-sensor method also include an inability to assess certain movements or components of criteria. Predominantly, this was because there was no sensor in or on the object, such as the ball or the wall. While IMU sensors can capture the motion of the body segments, they cannot capture interactions with external objects. This particularly applied to the object control skills. For example, in the catch, the key criteria of “catch ball with hands only” could not be assessed—because the ball was not able to be tracked. In the kick, two of the criteria could be assessed, but two could not: “non-kicking foot close to ball” and “use instep or inside of preferred foot to kick ball—not toe.” Similarly, two of the four criteria of the overhand throw could not be assessed, one of these due to lack of data from an external object (i.e., rotates hip and shoulders to a point where the non-throwing side faces the wall). This is a significant omission of important data required for comprehensive evaluation of skills. Thus, measurement of the object control subset of the TGMD-3 may be comprised by IMU-based systems. In comparison, manual assessment shows acceptable levels of inter-rater reliability for the overhand throw, but not for the catch and kick. For intra-rater reliability, acceptable values were present for the catch and overhand throw, but not the kick. Hence, both systems of assessment have challenges in terms of the object control skills.

In addition to non-measurable skills or skill criteria, another limitation of the simplified sensor method is inaccuracy leading to misclassification; for example, false positives (i.e., a student assessed as competent in a particular skill when in fact they were not) or false negatives (i.e., a student assessed as not yet competent in a particular skill when in fact they were). Why were certain skill criteria misclassified? The accuracy was lower for the throw, jump and hop. For example, in the jump, criterion 2 (i.e., “arms reaching forcefully above the head”) had no false rejections, whereas criteria 1, 3, and 4 had several false rejections, meaning that a good performance was classified as poor. However, criteria 2 of the jump did have several false acceptances, and in four cases a poor performance was classified as accurate. In such a small sample size, these misclassifications may be a concern and this level of “noise” in the data indicates that the accuracy is affected. However, it is important to note that misclassification of skill performance can also occur with manual assessment.

## Implications and Solutions

The level of potential misclassification with the sensor method may reduce the feasibility of the method, as inaccurate results make the method less appealing to teachers, who may then need to fall back on manual methods which they may perceive as more accurate.

The seven TGMD-3 skills tested (i.e., jump, hop, skip and sidestep, catch, throw, and kick) are commonly used in the Australian physical education curriculum. The remaining six motor skills in the TGMD-3 are the run, gallop, leap, two-handed strike, one-handed strike and stationary dribble. Extrapolating from the results of the seven skills tested in the simplified method, it might be expected that the other six skills may also be impacted by localization, in particular the object control skills, as these relate to an external object that may not be able to be picked up by IMUs as placed on a person. As such, additional parameters may be needed for criteria within those skills to inform how a performance is classified. For example, a compromise here may be a hybrid approach to assessment, where the skill and skill criteria that can be accurately assessed by IMU are automated, and the additional skill criteria assessed by other means (video or manual assessment). Potentially, an addition of one or two additional sensors may provide the depth of skill analysis and mitigate the omissions detected with the use of only four sensors. This may increase set up time, and dilute feasibility in terms of practicalities in a class, however, may provide the additional skill information. Research in the sports field is showing it is possible to implant sensors in objects to detect movement of the object, see for example, in basketball. So this could be the way of the future in motor competence assessment in children. Future research should test the real-word application of IMUs by teachers in schools and potential new ways to embed sensors.

## Conclusions

Although there are major advantages of using IMUs for motor competence assessment, there are also significant disadvantages with the present methods. Reducing the number of sensors to four creates a highly feasible method, but the results are incomplete. This enhanced feasibility and ease of use may lead to greater uptake, applicability, and use in real-world settings, particularly physical education, potentially leading to improved measurement at a population level, greater chance of targeted intervention and ultimately improved motor competence. However, some of the TGMD skill criteria were not entirely assessed in the context of machine learning, such as the fine detail of catching a ball in the hands. These gaps in the results reduce the quality of the data, and therefore may reduce its usefulness in detecting motor skill competence. This reduced accuracy may make the method less appealing to teachers—even though manual assessment of skill also has issues with reliability. Thus, while machine learning may enhance in-field application and feasibility of the TGMD, objective or automated implementation via sensor wear may not satisfy all assessment criteria. Could a compromise be struck regarding accuracy vs. feasibility? The question remains—how best to balance precision with practicality? Currently, motor skill competence is rarely conducted in schools, and skill errors are seldom remediated. Therefore, although IMUs may have some limitations, is this a case of something being better than nothing?

## Data Availability Statement

The raw data supporting the conclusions of this article will be made available by the authors, without undue reservation.

## Ethics Statement

The studies involving human participants were reviewed and approved by Deakin University ethics and the Department for Education and Training approved this research. Written informed consent to participate in this study was provided by the participants' legal guardian/next of kin.

## Author Contributions

All authors made substantial contributions to the conception and design of the work, contributed to the analysis or interpretation of data for the work, and assisted with drafting the work or revising it critically for important intellectual content. All authors have provide approval for publication of the content and agree to be accountable for all aspects of the work in ensuring that questions related to the accuracy or integrity of any part of the work are appropriately investigated and resolved.

## Conflict of Interest

The authors declare that the research was conducted in the absence of any commercial or financial relationships that could be construed as a potential conflict of interest.

## Publisher's Note

All claims expressed in this article are solely those of the authors and do not necessarily represent those of their affiliated organizations, or those of the publisher, the editors and the reviewers. Any product that may be evaluated in this article, or claim that may be made by its manufacturer, is not guaranteed or endorsed by the publisher.
